# Radiographic measurements for the prediction of dysphagia after occipitocervical fusion: a systematic review

**DOI:** 10.1007/s00701-023-05509-6

**Published:** 2023-02-14

**Authors:** Charles Tatter, Victor Gabriel El-Hajj, Alexander Fletcher-Sandersjöö, Erik Edström, Adrian Elmi-Terander

**Affiliations:** 1 Department of Clinical Neuroscience, Karolinska Institutet, Stockholm, Sweden; 2 Capio Spine Center Stockholm, Löwenströmska Hospital, Upplands Väsby, Sweden

**Keywords:** Occipitocervical fusion, Craniovertebral junction, Craniocervical junction, Dysphagia, Radiographic parameters, Sagittal radiographic angles

## Abstract

**Background:**

Occipitocervical fusion (OCF) is a procedure performed for multiple upper cervical pathologies. A common postprocedural complication of OCF is dysphagia, which has been linked to the narrowing of the pharyngeal space due to fixation in a hyper-flexed angle. Postoperative dysphagia is linked to reduced quality of life, prolonged hospital stay, aspiration pneumonia, and increased mortality. This has led to investigations of the association between sagittal radiographic angles and dysphagia following OCF.

**Methods:**

A systematic review of the literature was performed to explore the current evidence regarding cervical sagittal radiographic measurements and dysphagia following OCF. A search strategy was carried out using the PubMed, Embase, and Web of Science databases from their dates of inception until August 2022. Only original English-language studies were considered. Moreover, studies had to include the correlation between dysphagia and at least one radiographic measurement in the sagittal plane.

**Results:**

The search and subsequent selection process yielded eight studies that were included in the final review, totaling 329 patients in whom dysphagia had been assessed and graded. The dysphagia score by Bazaz et al. (*Spine* 27, 22:2453–2458, 2002) was used most often. The pooled incidence of dysphagia, in the early postoperative period, was estimated at 26.4%. At long-term follow-up (range: 17–72 months), about one-third of patients experienced resolution of symptoms, which resulted in a long-term post-OCF dysphagia incidence of 16.5%. Across the studies included, six different radiographic parameters were used to derive several measures which were repeatedly and significantly associated with the occurrence of dysphagia.

**Conclusions:**

The high incidence of postoperative dysphagia following OCF warrants close monitoring of patients, especially in the short-term postoperative period. These patients may be assessed through standardized tools where the one by Bazaz et al. was the most commonly used. Moreover, there are several radiographic measurements that can be used to predict the occurrence of dysphagia. These findings may serve as a basis for strategies to prevent the occurrence of dysphagia after OCF.

**Supplementary Information:**

The online version contains supplementary material available at 10.1007/s00701-023-05509-6.

## Background

Occipitocervical fusion (OCF) is a widely used treatment for instability in the craniocervical junction (CCJ). Instability in this region may be caused by trauma or pathologies such as infection, tumors, or arthritis [15, 16, 19]. The CCJ is a complex anatomical region that includes the atlanto-occipital and the atlanto-axial joints [14, 15] and is the most mobile segment of the spine [14]. Surgical fusion of the CCJ greatly reduces mobility of the upper cervical spine. Furthermore, an OCF may extend beyond the CCJ, and the lowest included segment may be lower cervical or upper thoracic, thereby affecting spine mobility across multiple joints.

Optimal perioperative positioning of the head is of great importance in OCF to attain joint angles that allow a neutral gaze without the need to strain the neck, which may create discomfort and pain [8, 22–24, 28]. An important complication to OCF is dysphagia, reported in 15.8 to 26.6% of cases [8, 11, 19, 25]. Postoperative dysphagia can lead to aspiration pneumonia, prolonged hospital stay, and increased mortality [3, 8, 9, 17, 22, 25]. In addition, dysphagia has been shown to greatly interfere with a patient’s quality of life [10, 11, 21, 25]. Sagittal malalignment at the CCJ has been postulated to be one of the main causes of dysphagia in these patients, as fusion in a hyper-flexed angle may cause a narrowing of the oropharyngeal space [7, 19, 22, 27]. Fixation of the subaxial cervical spine or across the cervico-thoracic junction may also influence the sagittal alignment and sagittal radiographic angles, subsequently causing dysphagia.

While there are multiple measurements of cervical sagittal radiographic angles, it remains unclear whether they are associated with dysphagia following OCF.

### Aim

The aim of this study was to systematically review the body of evidence regarding postoperative dysphagia in adult patients undergoing OCF, with a special focus on answering the following questions: what is the incidence of postoperative dysphagia after OCF? Can measurements of sagittal radiographic angles in the cervical spine predict postoperative dysphagia following OCF?

## Methods

### Search strategy

A systematic literature search was carried out in the electronic databases PubMed, Embase, and Web of Science from their dates of inception until August 2022. The search strategy comprised three major parts: anatomical region (occipitocervical junction), intervention (surgical fusion/fixation), and outcome (postoperative dysphagia). For each of the parts, a combination of relevant keywords was added to the final search strategy, with the help of simple Boolean operators (Supplementary file 1).

### Eligibility criteria

Studies targeted for inclusion in this review were those that examined the occurrence of postoperative dysphagia in adult patients (> 18 years) undergoing OCF. Only peer-reviewed original studies written in English were assessed for eligibility. Studies had to provide data on both postoperative incidence of dysphagia and the association to cervical sagittal radiographic angles. Reviews, case reports, and publications other than original research articles were excluded (Table 1).

**Table 1:** Inclusion and exclusion criteria

Inclusion	Exclusion
• Peer-reviewed original articles • Written in English • Surgically adult patients (≥ 18 years) • Patients treated with OCF • At least one radiographic angle measurement reported • Reported correlation between angle and occurrence of dysphagia	• Case reports • Case series where patients who did not develop dysphagia were excluded • Review articles • Editorials, letters, comments

### Data extraction, synthesis, and risk of bias assessment

Data extraction was performed on a predefined spreadsheet that included categories such as author information, sample size, follow-up time, dysphagia incidence, radiographic parameters studied, and associated correlations. Data analysis and subsequent narrative synthesis were conducted according to the Cochrane recommendations [13]. Risk of bias assessment using the Newcastle-Ottawa Scale (NOS) [6] revealed moderate to low risk of bias across all included studies.

## Results

### Study selection

**Fig. 1:**
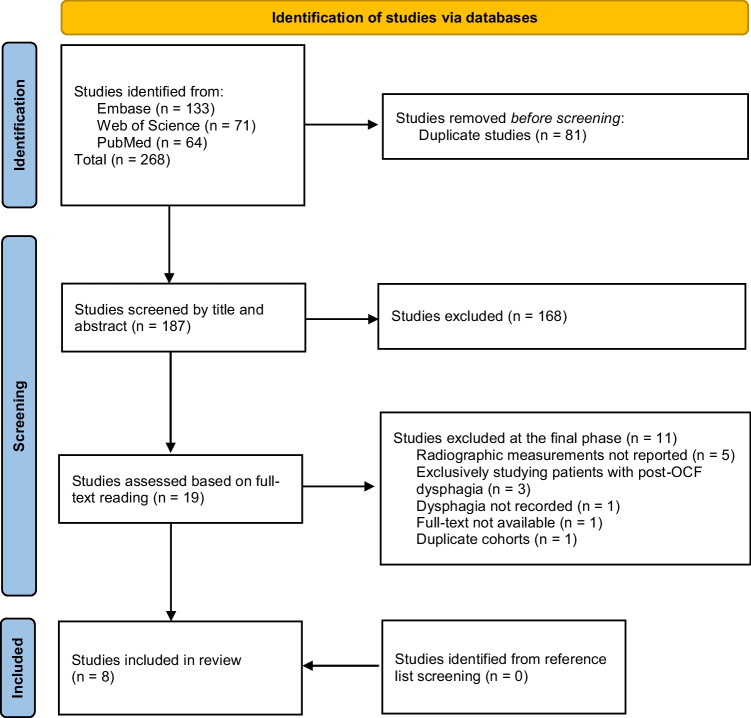
PRISMA flow chart

The search strategy applied to the three databases, as of August 2022, yielded 268 studies (Fig. 1). After manual identification and removal of duplicates, two independent and blinded reviewers (C.T. and A.F.-S.) were assigned the task of screening the remaining 187 studies based on titles and abstracts. This first step resulted in 19 studies. Subsequently, the full texts of the 19 studies were assessed for definitive inclusion by the same reviewers who were blinded from each other. Only studies adhering to the set of predefined criteria remained. During the finalization of the inclusion process, conflicts were resolved by team discussion and consultation with a third reviewer (A.E.-T.), as needed. Reference lists of the included studies were also screened for additional studies that could have been missed. Exclusions at the final step occurred for the following reasons: radiographic measurements not reported (*n* = 5), studies exclusively studying patients with postoperative dysphagia (*n* = 3), dysphagia not reported (*n* = 1), duplicate cohorts (*n* = 1), and full text not available (*n* = 1). This systematic review was performed in accordance with the PRISMA 2020 guideline (Supplementary file 2).

### Definition of postoperative dysphagia

Eight studies were included in this review (Table 2). The assessment and grading of postoperative dysphagia varied among the included studies. Three studies did not use standardized tools to assess patients with reported dysphagia. Two methods were used in the remaining five studies: (1) the dysphagia score by Bazaz et al. (*n* = 4) [1] (Table 2) and (2) the Functional Oral Intake Scale (FOIS; *n* = 1) by Crary et al. [5] (Table 3).

**Table 2:** The dysphagia classification grade by Bazaz et al. [1]

Severity of dysphagia	Difficulty swallowing liquid	Difficulty swallowing solids
None	None	None
Mild	None	Rare
Moderate	None or rare	Occasional
Severe	Present	Frequent

**Table 3:** The Functional Oral Intake Scale (FOIS) [5]

Severity level	Description
Level 1	Nothing by mouth
Level 2	Tube-dependent with minimal attempts of food or liquids
Level 3	Tube-dependent with consistent oral intake of food or liquids
Level 4	Total oral diet of a single consistency
Level 5	Total oral diet with multiple consistencies but requiring special preparations or compensations
Level 6	Total oral diet with multiple consistencies but with specific food limitations
Level 7	Total oral diet with no restrictions

The most commonly used dysphagia assessment tool by Bazaz et al. grades patients’ experience of swallowing difficulty from none to severe, where severe is defined as frequent difficulty swallowing the majority of foods [1]. The assessment tool FOIS, originally designed to evaluate the functional level of oral intake in stroke patients, is a 7-grade scale that ranges from no oral intake of food or liquids (1) to a complete diet with no restrictions (7) [5].

### Incidence of postoperative dysphagia

The incidence of postoperative dysphagia in the included studies ranged from 17.2 to 35% and from 3.8 to 25%, at short-term (< 6 months) and long-term follow-up (12–72 months), respectively.

Only one study did not report long-term follow-up data. Notably, resolution of symptoms at long-term follow-up was seen only in studies using self-reported swallowing difficulties rather than a standardized assessment tool. The studies are presented chronologically (Table 4).

**Table 4:** Summary of the eight included studies

Date and authors	Study design (*n* = sample; *FU* = mean *FU* in months)	Indications for surgery	Dysphagia at short-term follow-up (%)	Dysphagia at long-term follow-up (%)	Dysphagia assessment tool	Measured angles	Lowest fixation level (*n*)
2022 Zou et al. [30]	Retrospective cohort study (*n* = 40; *FU* = 72)	• RA • Atlantoaxial subluxation • Basilar invagination • Non-union fracture • Klippel-Feil syndrome	10 (25%)	10 (25%)	Dysphagia classification grade by Bazaz et al.	• O-C2a • O-C3a • O-Da • Oc-Axa • nPAS	• ≤ C3 (23) • > C3 (17)
2021 Wang L et al. [26]	Retrospective cohort study (*n* = 98; *FU* = 56.4)	• RA • Atlantoaxial subluxation • Basilar invagination • Congenital atlas • Odontoid deformity • Non-union fracture	26 (26.5%)	24 (24.5%)	Dysphagia classification grade by Bazaz et al.	• ADI • O-C2a • O-EAa • C2Ta • C2-C7a • PIA • nPAS	NA
2021 Miyagi et al. [18]	Retrospective cohort study (*n* = 20; *FU* < 1)	• RA • Cervical spondylosis • Trauma • Retro-odontoid pseudotumor • Other/non-specified	7 (35.0%)	NA	FOIS	• O-C2a • C2-C6a • PIA • nPAS	NA
2019 Chen et al. [4]	Retrospective cohort study (*n* = 22; *FU* = 17.2)	• RA • Non-union odontoid fracture • Basilar invagination	4 (18.2%)	2 (9.1%)	Dysphagia classification grade by Bazaz et al.	• O-C2a • C2Ta • O-EAa • nPAS	• C2 (17) • C3 (3) • C4 (2)
2018 Wang X. et al. [27]	Mixed retro- and prospective cohort study (*n* = 78; *FU* = 56.5)	• Atlantoaxial dislocation • Basilar invagination • O-C1 assimilation • Chiari • Klippel-Feil syndrome	19 (24.4%)	3 (3.8%)	Dysphagia classification grade by Bazaz et al.	• O-C2a • C2-C7a	NA
2018 Meng et al. [17]	Retrospective cohort study (*n* = 34; *FU* = 29.9)	• Atlantoaxial dislocation • Basilar invagination • Oncologic • Atlas deformity	6 (17.6%)	4 (11.8%)	No standardized classification system	• O-C2a • C2-C7a • ADI • nPAS	• 3 (18) • C4 (12) • C5 (4)
2017 Kaneyama et al. [11]	Retrospective cohort study (*n* = 38; *FU* = 46.8)	• RA • Atlantoaxial subluxation • Cerebral palsy • Tuberculous spondylitis • Klippel-Feil syndrome • Post-op. deformity	10 (26.3%)	6 (15.8%)	No standardized classification system	• O-C2a • PIA • PAS	NA
2009 Miyata et al. [19]	Retrospective cohort study (*n* = 29; *FU* = NM)	• RA • O-C1 assimilation • Oncologic • Other/non-specified	5 (17.2%)	4 (13.8%)	No standardized classification system	• O-C2a	• C2 (12) • C3 (6) • C4 (4) • C5 (2) • C7 (2) • Th1 (1) • Th2 (1) • Th4 (1)

*RA*, rheumatoid arthritis, *FOIS*, Functional Oral Intake Scale, *NA*, not available, *O*, occipital, *C*, cervical, *Th*, thoracic

Zou et al. (2022) studied 40 patients treated with OCF. Using the dysphagia score by Bazaz et al., the authors identified ten patients (25%) with postoperative dysphagia in the immediate postoperative period. None of the patients experienced total resolution of symptoms even at later follow-ups (average: 72 months) [30].

Wang et al. (2021) conducted the largest study in this review. They included 98 patients that had undergone OCF, of whom 26 (27%) reported postoperative dysphagia. At their last follow-up (average: 56.4 months), only two patients had recovered [26]. The grade of dysphagia was assessed through clinic or telephone interviews with the help of the dysphagia score by Bazaz et al.

In their cohort study, Miyagi et al. (2021) presented 22 patients treated with OCF. The FOIS was used to assess dysphagia during the first week after surgery. A FOIS score of 1–6 was classified as dysphagia and 7 as non-dysphagia. Seven of the 22 patients (32%) had reportedly developed dysphagia during the first week. Unfortunately, long-term follow-ups were not provided [18].

Chen et al. (2019) reported four postoperative cases of dysphagia among 22 patients who underwent OCF procedures. However, only two of the dysphagia cases reportedly persisted at last follow-up (average: 17.9 months) [4]. Assessments were made at the outpatient clinic or through telephone interviews using the dysphagia score by Bazaz et al.

Wang et al. (2018) reported 78 patients that were treated with OCF, of whom 19 (24%) reported dysphagia in the short-term postoperative period, but at last follow-up (average: 56.5 months), only three of these patients reported persisting dysphagia [27]. Assessments were made at the outpatient clinic or through telephone interviews using the dysphagia score by Bazaz et al.

Meng et al. (2018) conducted a study on 34 patients that had undergone OCF. There were six cases of dysphagia after 2 weeks, decreasing to four cases at the last follow-up (average: 29.9 months). The first six cases were found by retrospective analysis of medical records, while at the last follow-up, the patients were assessed at the outpatient clinic or through telephone interviews. No standardized assessment tools were used to grade dysphagia, and patients were defined as having dysphagia if they complained of swallowing difficulties [17].

Kaneyama et al. (2017) conducted a study on 38 patients that had been treated with OCF. At 1 week postoperatively, there were ten patients that had developed dysphagia. At the last follow-up (average: 46.8 months), four patients had complete symptom resolution. No standardized assessment tools were used to assess patients reporting dysphagia. Instead, their condition was tested and verified by fiberoptic esophagoscopy [11].

Miyata et al. (2009) reported five cases of dysphagia among 29 patients who underwent OCF. The cases were identified by retrospective review of medical records at the time of postoperative hospitalization. After 7 months, one of the patients experienced a resolution of symptoms, while the rest of the patients had persistent dysphagia. No standardized assessment tools were used to assess patients with reported dysphagia and the follow-up time was not defined [19].

### Sagittal radiographic measurements and OCF angles

**Fig. 2:**
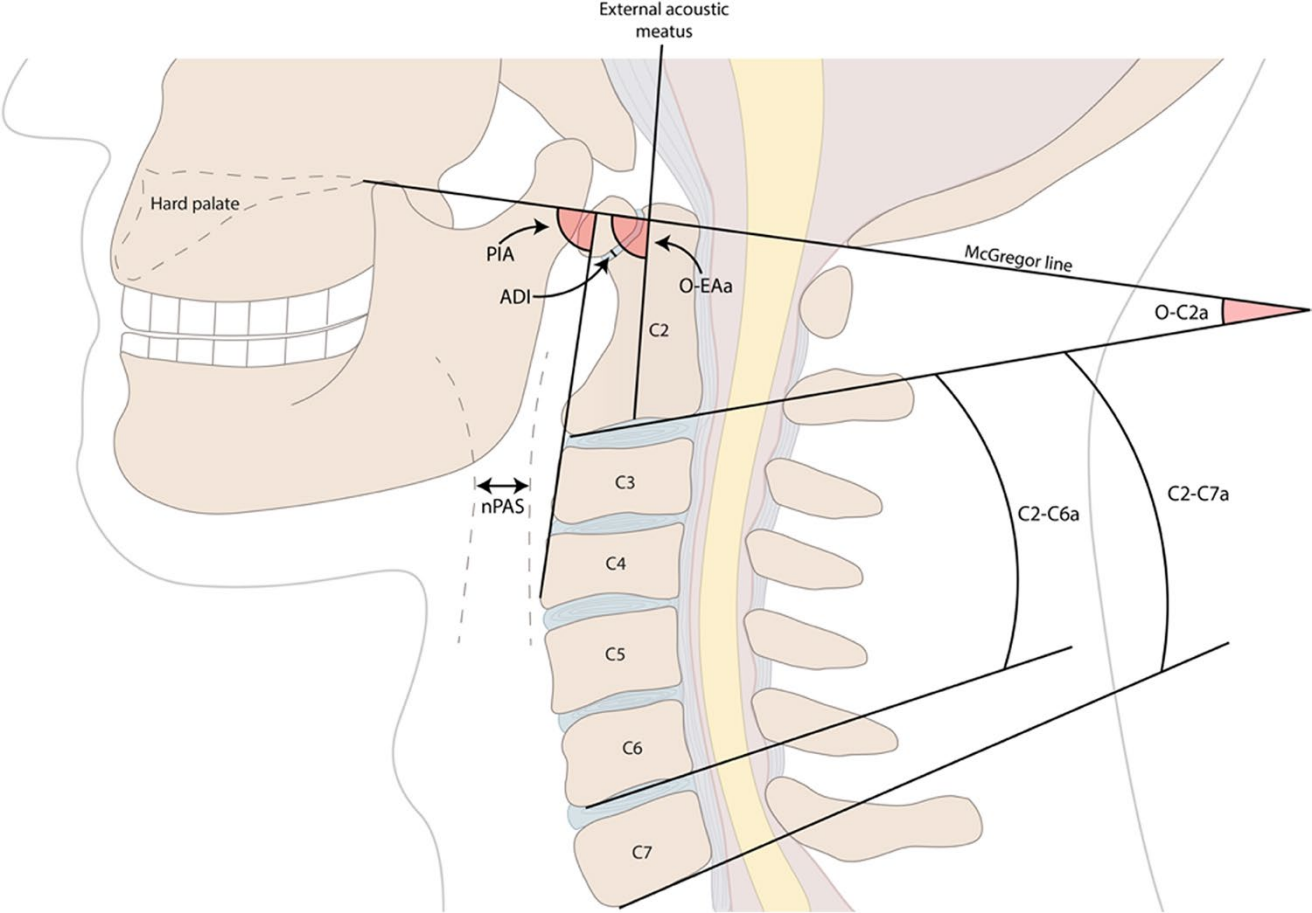
The radiographic measurements and angles most commonly reported in relation to OCF and dysphagia

The extent of the OCF fixation was reported in 4 studies. The fixations extended from the occiput to C2 in the shortest and from occiput to Th4 in the longest constructs. Moreover, a total of 12 different sagittal radiographic angles were measured on adult patients undergoing OCF across studies (Fig. 2). These angles were reported in a varying number of studies (range: 1–8) and are presented in order of frequency.

- **O-C2a**: The O-C2 angle (O-C2a) is defined as the angle between the McGregor line (the line connecting the posterior border of the hard palate and the most caudal portion of the occipital curve) and the inferior endplate line of C2. This angle was the one most commonly reported (*n* = 8 studies) [4, 11, 17–19, 26, 27, 30].
- **nPAS**: The narrowest oropharyngeal airway space (nPAS) is the narrowest anterior-posterior diameter of the oropharynx between the tip of the uvula and the tip of the epiglottis. This angle was the second most common to be studied (*n* = 5) [4, 17, 18, 26, 30].
- **PIA**: The pharyngeal inlet angle (PIA) is defined as the angle between the McGregor line and the line that links the center of the C1 anterior arch and the apex of the cervical sagittal curvature (*n* = 3) [11, 18, 26].
- **C2-C7a**: The C2-C7 angle (C2-C7a) is the angle formed between the inferior endplate lines of C2 and C7 (*n* = 3) [17, 26, 27].
- **C2Ta**: The C2 tilting angle (C2Ta) is formed by the inferior endplate of C2 and EA-line (*n* = 2) [4, 26].
- **O-EAa**: The occipital and external acoustic meatus-to-axis angle (O-EAa) is defined as the angle formed by the McGregor line and the line which connects the midpoint of the external acoustic meatuses and the midpoint of the inferior endplate of C2—also known as the EA-line (*n* = 2) [4, 26].
- **ADI**: The atlantodental/atlas-dens interval (ADI) is the distance between the back edge of the anterior arch of the atlas and the front edge of the odontoid process (*n* = 2) [17, 26].
- **C2-C6a**: The C2-C6 angle (C2-C6a) is the angle between the inferior endplate lines of C2 and C6 (*n* = 1) [18].
- **PAS**: The anteroposterior diameter of pharyngeal space (PAS) was measured at the level just cranial to the epiglottis (*n* = 1) [11].
- **O-C3a**: The O-C2 angle (O-C2a) is defined as the angle between the McGregor line and the inferior endplate of C3 (*n* = 1) [30].
- **O-Da**: The occipito-odontoid angle (O-Da) is defined as the angle formed by the McGregor line and the posterior longitudinal line of the C2 vertebra (*n* = 1) [30].
- **Oc-Axa**: The occipital to axial angle (Oc-Axa) was defined as the angle between the line connecting the occipital protuberance and the most caudal point on the midline occipital curve and the posterior longitudinal line of the C2 vertebra (*n* = 1) [30].

#### Correlations between radiographic angle measurements and postoperative dysphagia

Only measurements that had been studied in at least two of the included studies and associated with a significant correlation to dysphagia in at least one were regarded in the analysis. Six angles, which were used to calculate 13 different predictor variables, met these criteria and were included (Table 5).

**Table 5:** Sagittal radiographic angles in dysphagia groups compared to non-dysphagia groups, for measurements assessed in no less than two studies with at least one showing significant results

Studies	Post-O-C2a	∆O-C2a	Pre-PIA	Post-PIA	∆PIA	Post-O-EAa	∆O-EAa	Pre-nPAS	Post-nPAS	∆nPAS (cm)	Post-C2-C7a	∆C2-C7a	∆C2Ta
Miyata [19]													
D (5)	NS	–13.4	-	-	-	-	-	-	-	-	-	-	-
ND (24)		+2.0											
*p*-value		(0.002)											
Meng [17]													
D (6)	–5.16	–9.68	-	-	-	-	-	NS	+0.92	–0.48	NS	+10.36	-
ND (28)	+6.79	+2.83							+1.43	–0.05		–2.65	
*p*-value	(0.02)	(0.01)							(< 0.01)	(< 0.001)		(0.024)	
Wang X. [27]													
D (19)	NS	–9.31	-	-	-	-	-	-	-	-	NS	NS	-
ND (59)		+1.43											
*p*-value		(0.001)											
Miyagi [19]													
D (7)	NS	NS	+92.1	NS	NS	-	-	+1.43	NS	NS	-	-	-
ND (13)			+101.5					+2.11					
*p*-value			(< 0.05)*					(< 0.05)					
Wang L. [26]													
D (26)	+0.07	–9.51	+96.80	+81.85	–13.95	+94.21	–10.11	NS	+0.69	-	+24.60	+4.11	NS
ND (72)	+7.86	+3.82	+90.06	+91.28	+1.22	+101.42	+0.10		+1.43		+18.00	–7.66	
*p*-value	(0.005)	(0.000)	(0.005)*	(0.000)	(0.000)	(0.000)	(0.000)		(0.000)		(0.038)	(0.001)	
Chen [4]													
D (4)	NS	–0.81	-	-	-	+94.43	NS	+1.2	+0.7	–0.5	-	-	–4.36
ND (18)		+9.67				+97.63		+1.72	+1.4	–0.32			–20.18
*p*-value		(0.000)				(0.010)		(0.007)	(0.000)	(0.019)			(0.001)
Kaneyama [11]													
D (6)	NS	-	-	75.3	-	-	-	-	-	-	-	-	-
ND (32)				90.3									
*p*-value				(0.019)									
Zou [30]													
D (10)	NS	–6.0	-	-	-	-	-	NS	+0.8	–0.4	-	-	-
ND (30)		+2.6							+1.5	+0.1			
*p*-value		(0.001)							(<0.001)	(0.003)			

All variables are measured in degree (°), except when otherwise stated

*Significance was in opposite directions

*D* Dysphagia group, *ND* non-Dysphagia group, *NS* not significant (*p* > 0.05), ∆ post- and pre-operative difference

#### Correlations using preoperative radiographic angle measurements

The preoperative PIA was assessed in two studies with conflicting results. Miyagi et al. found a significantly lower preoperative PIA in the dysphagia group when comparing to the non-dysphagia group [18]. Wang et al. found the opposite, significantly associating a higher preoperative PIA to dysphagia [26]. Two studies found a significant association between a lower preoperative nPAS and dysphagia [4, 18]. Three other studies that tested the same hypothesis did not identify significant results [17, 26, 30]. The Oc-Axa was found to be significantly lower in patients who presented with postoperative dysphagia. However, this association was examined in only one study [30].

#### Correlations using postoperative radiographic angle measurements

All eight studies examined the association between lower O-C2a and the occurrence of dysphagia. While two studies established a significant association [17, 26], six studies did not [4, 11, 18, 19, 27, 30]. In addition, a significantly lower postoperative PIA was found in two of three studies using this measurement [11, 18, 26]. In the studies that considered the postoperative nPAS (*n* = 5) [4, 17, 18, 26, 30] and O-EAa (*n* = 2) [4, 26], these angles were found to be significantly lower in patients with dysphagia. In one of three studies, a higher C2-C7a was associated with dysphagia [17, 26, 27].

#### Correlations using the difference (∆) between preoperative and postoperative radiographic angle measurements

Six studies demonstrated significantly lower ∆O-C2a in patients that had developed dysphagia [4, 17, 19, 26, 27, 30], while only one study reported no significant association [18]. The ∆PIA and ∆EAa measures were assessed in two studies. Only one of the two studies could find a significant association between a lower ∆PIA [26] or a lower ∆EAa [26] and the development of dysphagia. The ∆nPAS was measured in four different studies [4, 17, 18, 30]. All but one identified a significant association between lower ∆nPAS and the occurrence of dysphagia [18]. The alternative metric ∆nPAS%, measured as the (postoperative nPAS − preoperative nPAS)/preoperative, was significantly associated with dysphagia in the only study where it was used [26]. The ∆C2-C7a was studied in three studies and found to be significantly higher for dysphagia patients in two [17, 26]. Similarly, the ∆C2Ta was studied in two studies and significantly higher for dysphagia patients in one [4]. Lower ∆O-C3a, ∆O-Da, and ∆Oc-Axa were significantly associated with dysphagia; however, the results were only validated by one study [30].

## Discussion

### Overview

The aim of this study was to systematically review the literature regarding possible correlations between sagittal radiographic angles and dysphagia after OCF. The database search as well as the selection process yielded eight studies with a combined total of 329 patients. At the short-term follow-up, 26.4% of these patients had reportedly developed dysphagia. At long-term follow-ups, which varied between 17 and 72 months among the studies, this number dropped to 16.5%. Only one study reported no patients with symptom resolution at the last follow-up, although the severity of the symptoms was reduced [30]. Notably, the definition of short- and long-term follow-up, as well as the definition of dysphagia, varied among studies. Wang et al. considered a dysphagia persisting less than 1 month postoperatively to be the result of intubation and hence disregarded these symptoms [26]. Conversely, Miyagi et al. exclusively considered patients reporting dysphagia within the first week after surgery [18]. This highlights the heterogeneity in defining postoperative dysphagia and the need to establish a common definition.

Heterogeneity was also seen in the methods of assessment and grading of dysphagia. The most frequently used assessment tools were the dysphagia score, designed by Bazaz et al. in 2002 [1], and the FOIS, originally intended to assess stroke patients [5]. Surprisingly, several authors did not use standardized assessment tools at all. Although no studies in this review reported its use, the Dysphagia Short Questionnaire (DSQ) is a well-established tool for the assessment and grading of dysphagia symptoms. The DSQ is a scoring system of 0–18 where low numbers indicate mild symptoms and a higher score more prominent symptoms. The points are divided among five questions covering different aspects of dysphagia (ability to swallow, incorrect swallowing, lump feeling, involuntary loss of weight, and pneumonia) [20].

Many pre- and postoperative sagittal radiographic angles have been suggested to predict postoperative dysphagia after OCF. The most frequently examined angle was the O-C2a, which is one of the most well-established angles in the field of cervical biomechanics [29]. The examined studies found no use for the preoperative O-C2a in the prediction of postoperative dysphagia. The postoperative O-C2a was significantly associated with dysphagia in only two of the eight studies. In contrast, the ∆O-C2a does seem to play a role, as a lower ∆O-C2a was significantly associated to dysphagia in six of seven studies. These findings may support the hypothesis that dysphagia results from a decrease in O-C2a after surgery, leading to a narrowing of the oropharyngeal space. Lending credibility to this hypothesis, several studies have shown significant differences in the postoperative PAS and ∆nPAS between the dysphagia and non-dysphagia groups [4, 17, 26, 30]. Besides O-C2a and PAS, other promising predictors of dysphagia were discovered in this review. The O-EAa and PIA measure a similar angle reflecting the degree of flexion at the upper cervical levels and small angles may be taken to represent a narrowing of the oropharyngeal passages. The postoperative O-EAa was significantly associated with dysphagia in the two studies where this parameter was measured. Similarly, PIA could also predict dysphagia. Both the pre- and postoperative PIA were significantly lower in patients experiencing dysphagia after OCF [11, 26]. A recent study found several radiographic angles to be related to measures of health-related quality of life after OCF surgery. Restoration of O-C2a and PIA to normal physiologic ranges were found to improve the health-related quality of life through an impact on breathing, swallowing, and horizontal gaze [12].

In summary, based on the findings of this review, we recommend that patients undergoing OCF should be postoperatively assessed for dysphagia, as almost 35% of patients undergoing OCF at short-term and 25% of patients at long-term were found to develop this complication. A standardized and validated assessment tool, such as the Bazaz Dysphagia Score, FIOS, or DSQ, should be used for this purpose.

Almost half of the patients presenting with dysphagia during the short-term period following the surgery experienced improvements or total resolution of symptoms. Moreover, measurement of cervical sagittal radiographic angles pre-, intra-, and postoperatively may help in predicting or avoiding dysphagia. As seen in this review, preoperative radiographic parameters played a smaller role in predicting postoperative dysphagia when compared to postoperative angle measurements or differences between pre- and postoperative measurements. The radiographic parameters that exhibited the strongest correlations to postoperative dysphagia were a lower ∆O-C2a, postoperative PIA, postoperative O-EAa, post-nPAS, and ∆nPAS, as well as a higher ∆C2-C7a. However, specific thresholds for dysphagia were poorly studied, and conclusions as to what specific angles to adhere to for the avoidance of dysphagia were not made. Future studies should be focused on determining practical guidelines to assist the performance of safe OCF to minimize the risk of postoperative dysphagia.

### Strengths and limitations

This is the first systematic review on the incidence and radiographic predictors of dysphagia after OCF. As for all systematic reviews, the limitations inherently stem from the studies included. Our systematic review is hampered by the small number of studies included, the small sample sizes, and the retrospective nature of the studies. The lack of prospective and randomized trials severely limits the strength of evidence that can be achieved. Moreover, the large heterogeneity with respect to indications for surgery, follow-up times, and, most importantly, the definition of dysphagia did not allow for a quantitative or statistical analysis. In addition, most of the studies included in this review originated from East-Asia. This affects the external validity of our results, but also highlights a gap in the research field that requires attention.

## Conclusion

This review aimed to summarize the current evidence regarding the occurrence of dysphagia after OCF and its association to radiographic measurements. Following OCF, 26.4% of patients reportedly experienced dysphagia. This number subsequently dropped to 16.5% at long-term follow-up, indicating that more than one-third of the patients with postoperative dysphagia may experience resolution of symptoms. Dysphagia can be assessed and graded with available standardized assessment tools. This systematic review also identified promising radiographic predictors of postoperative dysphagia, such as lower ∆O-C2a, postoperative PIA, postoperative O-EAa, post-nPAS, and ∆nPAS, as well as a higher ∆C2-C7a. Most of the conclusions drawn rely on level 2b evidence with a moderate to low risk of bias [2]. A larger number of validating studies with higher design quality is required to validate the associations and strengthen the certainty of evidence surrounding the topic.

## Supplementary Information

Below is the link to the electronic supplementary material.

Supplementary file1 (DOCX 67 KB)

Supplementary file2 (DOCX 131 KB)

## Data Availability

Not applicable.
