# Supplementary material for: Radiographic measurements for the prediction of dysphagia after occipitocervical fusion: a systematic review
Source: Acta Neurochir (Wien). 2023 Feb 14;165(5):1161–70. doi: 10.1007/s00701-023-05509-6 (PMC10140007; doi:10.1007/s00701-023-05509-6)
Supplement: Supplementary file 1 — Supplementary file1 (DOCX 67 KB) [file 701_2023_5509_MOESM1_ESM.docx]

*Table 1. Major concepts and keywords*

| Concept | Atlanto-occipital joint | Surgical fusion/fixation | Postoperative dysphagia |
| --- | --- | --- | --- |
| Keywords | Atlanto-occipital, atlantooccipital, occipito-cervical, occipitocervical, c0 | Fusion, Fixation, surgery | Dysphagia, swallow, deglutition disorder |

*Table 2. Search phrase, filters added, and results*

| Database | Search phrase | Filters added | # Results |
| --- | --- | --- | --- |
| PubMed | (Atlanto-occipital joint*[Title/Abstract] OR atlanto-occipital*[Title/Abstract] OR atlantooccipital*[Title/Abstract] OR occipto-cervical*[Title/Abstract] OR occipitocervical*[Title/Abstract] OR c0*[Title/Abstract]) AND (fusion*[Title/Abstract] OR fixation*[Title/Abstract] OR surgery*[Title/Abstract] ) AND (dysphagia*[Title/Abstract] OR swallow*[Title/Abstract] OR deglutition disorder*[Title/Abstract]) | - English only - Full text available | 57 |
| Embase | (atlanto-occipital OR atlantooccipital OR occipto-cervical OR occipitocervical OR c0) AND (fusion OR fixation OR surgery) AND (dysphagia OR swallow OR deglutition disorder) | - Only in English - With abstract - Article | 123 |
| Web of Science | (atlanto-occipital OR atlantooccipital OR occipto-cervical OR occipitocervical OR c0) AND (fusion OR fixation OR surgery) AND (dysphagia OR swallow OR deglutition disorder) | - Languages: English - Document types: Articles | 67 |
